# VTE Prophylaxis Quality Improvement of Service Users Data in Older Adult Mental Health Inpatient Wards in St Charles Hospital, CNWL NHS Trust

**DOI:** 10.1192/bjo.2024.445

**Published:** 2024-08-01

**Authors:** Shreena Thakaria

**Affiliations:** St Charles Hospital, London, United Kingdom

## Abstract

**Aims:**

To reach the target of 100% for VTE (venous thromboembolism) prophylaxis data submitted for all St Charles Older Adult inpatients.

**Methods:**

It was found at the start of the QI project, the service was at 63% (August 2023). I reviewed this data and discussed it with the ward managers of the older adult inpatient wards and implemented two PDSA cycles. I went through the ward list of service users and noted on the database who had an outstanding VTE prophylaxis check. From this, I then created a section for the nursing handovers to include whether each service user had their VTE prophylaxis forms filled in and whether VTE prophylaxis was appropriately prescribed. The wards have a weekly MDT meeting where this could be discussed and all staff could be reminded to document the VTE data on the trust data system. I rechecked the data two months later to see if the data had improved. Following this, I created a VTE poster to be distributed via email to ward staff and hung up in the ward doctors' offices to help educate staff on the importance of VTE prophylaxis. The statistics were rechecked two months later for further improvement.

**Results:**

At the start of the QI, it was found that the service was underperforming in reaching its target of 100% of the VTE prophylaxis data entry for all service users in older adult inpatient wards. After implementing the first PDSA cycle, the data increased to 84% compliance (October 2023 data). After implementing the second PDSA cycle, the data increased to 100% compliance (December 2023 data). The data showed both implementations had a significant impact on the data input and the target being reached. The new strategy has now been firmly placed into the team working pattern as a routine measurement and continues to be actively utilised.

**Conclusion:**

In an older adult inpatient ward setting with service users who have co-morbidities, reduced mobility and risk of dehydration from self neglect, it is vital they are assessed appropriately for VTE risk factors and prescribed the appropriate prophylaxis. Once this was highlighted to the ward staff and an easy system of the PDSAs were implemented, the team are now able to actively input the data and provide optimal care for the service users.

